# Supplementary material for: Can Extracts from the Leaves and Fruits of the Cotoneaster Species Be Considered Promising Anti-Acne Agents?
Source: Molecules. 2022 May 2;27(9):2907. doi: 10.3390/molecules27092907 (PMC9105355; doi:10.3390/molecules27092907)
Supplement: Supplementary file 1 [file molecules-27-02907-s001.zip › molecules-1704941-supplementary.pdf]

Table S1. High-resolution mass spectrometry (HR-MS) of [M-H]- ion and MS2 data.

Peak No.	Name of Compound	[M-H]-	MS2	Theoretical Mass [M-H]- (Da)	Experimental Mass [M-H]- (Da)	Δ ppm	Δ mDa	Elemental Composition
1	Mannitol	181	59,71,73,85,89,101b,113,119,163	181.07122	181.07118	0.22	-0.04	$C_6H_{13}O_6$
2	Gallic acid	169	69, 79, 81, 97, 124, 125b	169.0137	169.01373	0.18	0.03	$C_7H_6O_5$
3	3-*O*-Caffeoylquinic acid	353	135, 179, 191b	353.08726	353.08715	0.31	-0.11	$C_{16}H_{18}O_9$
4	Vanillic acid-hexoside	329	136, 151, 167b, 329	329.08726	329.08716	0.30	-0.10	$C_{14}H_{18}O_9$
5	Syringic acid hexoside	359	85, 121, 138, 153, 197b	359.09783	359.09779	0.11	-0.04	$C_{15}H_{20}O_{10}$
6	(+)-Catechin	289	109, 123, 125, 137, 165, 179, 203, 205, 245, 289b	289.07122	289.07117	0.17	-0.05	$C_{15}H_{14}O_6$
7	Procyanidin C-1	865	125, 161, 243, 287, 289, 407, 425, 575, 577, 695, 865b	865.19800	865.19813	0.15	0.13	$C_{45}H_{38}O_{18}$
8	Protocatechuic acid	153	109b, 153	153.01879	153.01868	0.72	-0.11	$C_7H_6O_4$
9	Procyanidin B-2	577	125, 289b, 407, 577	577.13461	577.13449	0.21	-0.12	$C_{30}H_{26}O_{12}$
10	5-*O*-Caffeoylquinic acid (chlorogenic acid)	353	179, 191b	353.08726	353.08719	0.20	-0.07	$C_{16}H_{18}O_9$
11	p-Hydroxybenzoic acid	137	92, 93, 136, 137b	137.02387	137.02377	0.73	-0.10	$C_7H_6O_3$
12	(-)-Epicatechin	289	109, 123, 125, 137, 151, 165, 179, 203, 205, 245, 289b	289.07122	289.07137	0.52	0.15	$C_{15}H_{14}O_6$
13	4-*O*-Caffeoylquinic acid	353	135, 173b, 179, 191	353.08726	353.08716	0.28	-0.10	$C_{16}H_{18}O_9$
14	Caffeic acid hexoside	341	97, 107, 135, 179b, 341	341.08726	341.08739	0.38	0.13	$C_{15}H_{18}O_9$
15	Caffeic acid	179	97, 107, 135b	179.03444	179.03453	0.50	0.09	$C_9H_8O_4$
16	Syringic acid	197	85, 121b, 138, 153, 181, 197	197.04500	197.04513	0.66	0.13	$C_9H_{10}O_5$
17	*p*-Coumaric acid	163	119, 162b	163.03952	163.03962	0.61	0.10	$C_9H_8O_3$
18	*o*-Coumaric acid	163	119, 162b	163.03952	163.03943	0.55	-0.09	$C_9H_8O_3$
19	Vanilin	151	136, 151b	151.03952	151.03942	0.66	-0.10	$C_8H_8O_3$
20	Ferulic acid	193	73, 89b, 106, 117, 133, 134, 179	193.05009	193.05021	0.62	0.12	$C_{10}H_{10}O_4$
21	3,5-Di-*O*-caffeoylquinic acid	515	191, 335, 353b, 515	515.11896	515.11891	0.10	-0.05	$C_{25}H_{24}O_{12}$

22	Querectin 3-*O*-β-D-(6″-*O*-α-L-rhamnosyl) glucoside (rutin)	609	151, 179, 301b, 463, 609	609.18195	609.18207	0.20	0.12	$C_{27}H_{30}O_{16}$
23	3-*O*-*p*-Coumaroylquinic acid	337	163, 173b	337.09235	337.09249	0.42	0.14	$C_{16}H_{18}O_{8}$
24	Quercetin 3-*O*-β-D-(2″-*O*-β-D-xylosyl)galactoside	595	151, 179, 301, 433b, 595	595.12992	595.12983	0.15	-0.09	$C_{26}H_{28}O_{16}$
25	5-*O*-*p*-Coumaroylquinic acid	337	163, 173b	337.09235	337.09221	0.42	-0.14	$C_{16}H_{18}O_{8}$
26	Quercetin-3-*O*-(2′′-*O*-xylosyl)galactoside	595	271,301b,435	595.12992	595.12999	0.12	0.07	$C_{26}H_{27}O_{16}$
27	Quercetin-3-*O*-gentiobioside	625	271,301b,463	625.14048	625.14038	0.16	-0.10	$C_{27}H_{29}O_{17}$
28	Vitexin-2′′-*O*-arabinoside	563	283,432b	563.14009	563.14001	0.14	-0.08	$C_{26}H_{27}O_{14}$
29	Apigenin-6,8-*C*-dicelobioside	593	325,386b,387	593.15065	593.15077	0.20	0.12	$C_{27}H_{29}O_{15}$
30	Vitexin-2′′-*O*-rhamnoside	577	283,432b	577.15574	577.15571	0.05	-0.03	$C_{27}H_{29}O_{14}$
31	Quercetin-3-*O*-glucoside (isoquercitrin)	463	151, 179, 192, 301, 461, 463b	463.08766	463.08749	0.37	-0.17	$C_{21}H_{20}O_{12}$
32	Quercetin 3-*O*-β-D galactoside (hyperoside)	463	151, 179, 255, 271, 301b	463.08766	463.08755	0.24	-0.11	$C_{21}H_{19}O_{12}$
33	Kaempferol 3-*O*-glucoside (astragalin)	447	284b,300	447.09274	447.09289	0.34	0.15	$C_{21}H_{19}O_{11}$
34	Quercetin 3-*O*-rhamnoside (quercitrin)	447	151, 243, 255, 271, 300b, 301, 447	447.09274	447.09281	0.16	0.07	$C_{21}H_{20}O_{11}$
35	Hesperidin	609	151, 179, 301b, 302, 609	609.18195	609.18181	0.23	-0.14	$C_{28}H_{34}O_{15}$
36	Naringin	579	145, 151, 271, 579b	579.17139	579.17131	0.14	-0.08	$C_{27}H_{32}O_{14}$
37	Biochanin-7-*O*-glucoside	445	132, 184, 212, 223, 239b, 240, 267, 268, 283b	445.11348	445.11357	0.20	0.09	$C_{22}H_{22}O_{10}$
38	Rosmarinic acid	359	73, 123, 133, 135, 161b, 179, 197, 359	359.0767	359.07681	0.31	0.11	$C_{18}H_{16}O_{8}$
39	Sinapic acid	223	59, 141, 148, 164b, 178, 208, 223	223.06065	223.06075	0.45	0.10	$C_{11}H_{12}O_{5}$
40	Cinnamic acid	147	129, 147b	147.04461	147.04468	0.48	0.07	$C_{9}H_{8}O_{2}$
41	Quercetin	301	151, 179b, 192, 209	301.03483	301.03499	0.53	0.16	$C_{15}H_{10}O_{7}$
42	Kaempferol	285	93, 97, 119b, 164, 285	285.03992	285.03984	0.28	-0.08	$C_{15}H_{10}O_{6}$

43	7-Methylkaempferol-4′-*O*-glucoside	461	298b,315	461.10839	461.10849	0.22	0.10	$C_{22}H_{21}O_{11}$
44	3′,4′-dihydroxy-6-methoxyflavone 7-*O*-rhamnoside	429	255,283b,400,401	429.11856	429.11853	0.07	-0.03	$C_{22}H_{21}O_{9}$
45	Apigenin 8-*C*-glucoside (vitexin)	431	283,311b,312,341	429.11856	429.11842	0.33	-0.14	$C_{21}H_{19}O_{10}$
46	Apigenin 7-*O*-glucoside	447	269b,270,431	431.09783	431.09797	0.32	0.14	$C_{21}H_{19}O_{10}$
47	Biochanin A 7-*O*-glucoside (sissotrin)	445	132,211,223,224,239b,240,267	445.11348	445.11355	0.16	0.07	$C_{22}H_{21}O_{10}$
48	5,7,2′,5′-tetrahydroxyflavanone 7-*O*-glucoside	449	286b,302	449.10839	449.10825	0.31	-0.14	$C_{21}H_{21}O_{11}$
49	5-Methylgenistein 4′-*O*-glucoside	445	255,283b,417,430	445.11348	445.11339	0.20	-0.09	$C_{22}H_{21}O_{10}$
50	Orbicularin	315	153b,271	315.07161	315.07149	0.38	-0.12	$C_{13}H_{15}O_{9}$
51	Caffeoylmalic acid	295	133,135,179b	295.0454	295.04533	0.24	-0.07	$C_{13}H_{11}O_{8}$
52	Prunasin	294	161b	294.09777	294.09771	0.20	-0.06	$C_{14}H_{16}NO_{6}$
53	Amygdalin	456	89,119,143,179,221,263,323b	456.15059	456.15047	0.26	-0.12	$C_{20}H_{26}NO_{11}$
54	7,8-dimethoxy-6-hydroxycoumarin	221	191,206b,207	221.045	221.04506	0.27	0.06	$C_{11}H_{9}O_{5}$
55	Cotonoate A	291	121,165,247b	291.15964	291.15975	0.38	0.11	$C_{17}H_{23}O_{4}$
56	Horizontoate A	263	148,164,219b	263.12834	263.12852	0.68	0.18	$C_{15}H_{19}O_{4}$
57	3,3',4'-tri-*O*-methylellagic acid	343	255,284,299b,329	343.0454	343.04554	0.41	0.14	$C_{17}H_{11}O_{8}$
58	Scopoletin	191	104,105,120,148b,176	191.03444	191.03439	0.26	-0.05	$C_{10}H_{7}O_{4}$
59	Arbutin	271	71,101,108b,109,113,161	271.08178	271.08175	0.11	-0.03	$C_{12}H_{15}O_{7}$
60	5-Methylgenistein	283	239b,255,269	283.06065	283.06053	0.42	-0.12	$C_{16}H_{11}O_{5}$
61	Eriodictyol	287	119, 135b, 227	287.05557	287.05577	0.70	0.20	$C_{15}H_{12}O_{6}$
62	Luteolin	285	133, 151, 175, 199, 285b	285.03992	285.03985	0.25	-0.07	$C_{15}H_{10}O_{6}$
63	Apigenin	269	117, 149, 151, 225, 269b	269.04500	269.04509	0.33	0.09	$C_{15}H_{10}O_{5}$
64	Biochanin	283	132, 184, 211, 212, 223, 239b, 240, 267, 268	283.06065	283.06055	0.35	-0.10	$C_{16}H_{12}O_{5}$
65	5,7,2′,5′-tetrahydroxyflavanone	287	151,162b,259	287.05557	287.05564	0.24	0.07	$C_{15}H_{11}O_{6}$
66	Naringenin	271	119,151b,187	271.06065	271.06061	0.15	-0.04	$C_{15}H_{11}O_{5}$
67	Horizontoate C	482	210,272b,288	482.42093	482.42097	0.08	0.04	$C_{29}H_{56}NO_{4}$

b – base peak
